# Emergency Department Feasibility of the Brief Test of Adult Cognition by Telephone (BTACT) for Research in Mild Traumatic Brain Injury

**DOI:** 10.1111/acem.70420

**Published:** 2026-09-29

**Authors:** David J. Barton, Charles F. Rudolph, Ekaterina K. Kalinina, Keshav Reddy, Natalee Gallo, Harry Morford, Xuemei Zeng, Thomas K. Karikari, Maria L. Pacella‐LaBarbara, Clifton W. Callaway, Amy K. Wagner

**Affiliations:** ^1^ Department of Emergency Medicine University of Pittsburgh School of Medicine Pittsburgh Pennsylvania USA; ^2^ Department of Emergency Medicine Florida Atlantic University Charles E. Schmidt College of Medicine Boca Raton Florida USA; ^3^ Department of Psychiatry University of Pittsburgh School of Medicine Pittsburgh Pennsylvania USA; ^4^ Safar Center for Resuscitation Research University of Pittsburgh Pittsburgh Pennsylvania USA; ^5^ Department of Physical Medicine & Rehabilitation University of Pittsburgh School of Medicine Pittsburgh Pennsylvania USA; ^6^ Department of Neuroscience University of Pittsburgh Pittsburgh Pennsylvania USA

Mild traumatic brain injury (mTBI) accounts for millions of emergency department (ED) visits annually, and many patients have neurocognitive sequelae including persistent symptoms and incomplete recovery for months after injury [1]. A major challenge for mTBI clinical care and research is measuring injury‐related cognitive dysfunction because mTBI symptoms are nonspecific, and instruments typically used to measure cognitive performance remain unvalidated in ED settings. Assessing cognitive dysfunction in the ED is pragmatically challenging because of ED‐specific pressures including time, space, and patient acuity. Optimized methods for cognitive testing in ED patients could enhance clinical recognition of mTBI and improve clinical research needed to study mTBI treatments.

The Brief Test of Adult Cognition by Telephone (BTACT) is one potential solution for an ED‐based cognitive test. The BTACT was developed from established neuropsychological tests and evaluates episodic memory, working memory, reasoning, verbal fluency, and executive function [2]. It can also be used via telephone or in‐person. The BTACT has been used in TBI cohorts [3, 4, 5] and shows convergent validity with established TBI cognition measures [3], but has not been applied in ED settings. The BTACT's minimal equipment requirements, assessment of multiple cognitive domains, and relatively brief administration time make it a potentially advantageous assessment for ED use.

Our primary goal was to assess the feasibility of BTACT administration for acute mTBI research purposes, including frequency of successful completion, testing time required for BTACT testing, and impact on ED length of stay. Secondly, we explored pilot data on BTACT associations with brain injury indicators including TBI symptom burden and TBI blood biomarker levels.

We conducted a prospective observational cohort study of adults with mTBI presenting for ED evaluation. We consecutively recruited patients from two level 1 trauma center EDs within one academic healthcare system. Our university's Institutional Review Board approved this research. Participants provided written informed consent. Included subjects were between the ages of 18–64 years, had a confirmed diagnosis of mTBI or concussion from the treating physician, within 7 days post‐injury, and discharged home from the ED. Exclusion criteria were positive head CT findings, prior history of TBI (including concussion) or neurological disease, and hospital admission, among others (Figure S1).

Research staff screened active ED patient lists during 0800–2000 h for eligible subjects based on chief complaint. Research staff confirmed that subjects met mTBI diagnostic criteria based on 2023 American Congress of Rehabilitation Medicine consensus criteria. After informed consent, participants underwent survey data collection, cognitive testing with the BTACT (except in the pre‐BTACT study phase), and blood sampling in the ED. We recorded ED length of stay (time of ED check‐in to time of discharge) and receipt of diagnostic head imaging or laboratory studies (as other contributors to length of stay) from electronic health records.

Participants self‐reported the Rivermead Post‐concussion symptoms Questionnaire (RPQ) score. RPQ is a well‐validated post‐concussive symptom assessment consisting of 16 questions evaluating symptom severity from 0 (not experienced at all) to 4 (a severe problem) [6]. Three questions assess cognition‐related symptoms (“forgetfulness, poor memory”, “poor concentration”, and “taking longer to think”) and were summed as an RPQ cognitive sub‐score.

After an initial (pre‐BTACT) study phase, we added the BTACT to the study protocol to better assess cognition. This allowed a natural comparison of metrics before and after BTACT introduction. The BTACT is a cognitive test battery used previously for mTBI in non‐ED settings [7], consisting of six subtests: Word List Immediate Recall (measures verbal learning via immediate recall of a 15‐word list), Word List Delayed Recall (verbal memory retrieval), Digits Backward (working memory span), Number Series (inductive reasoning), Animal Fluency (verbal fluency as an index of executive function), and Backward Counting (processing speed). Trained research coordinators administered the BTACT using standardized instructions [8]. Staff audio recorded examinations for grading purposes, which also provided assessment length of time.

Staff collected venous blood samples into K3EDTA collection tubes, centrifuged blood tubes at 1500× relative centrifugal force for 10 min, aliquoted plasma samples, and froze samples at −80°C. We batch assayed plasma using the Quanterix Simoa Neurology 4‐plex B assay, which measures concentrations of four protein biomarkers of brain injury: Glial Fibrillary Acidic Protein (GFAP), Ubiquitin C‐Terminal Hydrolase L1 (UCH‐L1), Neurofilament Light Chain (NfL), and Tau. Coefficients of variation were < 10%.

BTACT feasibility measures included the percentage of participants who successfully completed the test, test completion time, and difference in ED length of stay associated with BTACT administration. In secondary analyses, we quantified the relationship between BTACT composite scores and RPQ score sum, RPQ‐cognitive sub‐score, and TBI biomarker levels. Spearman correlation coefficients were used to assess correlation between continuous variables. Mann–Whitney *U* tests were used to compare continuous variables between groups. All tests were two‐tailed. *p* values < 0.05 were considered statistically significant. Statistical analyses were conducted using GraphPad Prism version 10.6.1 for Windows. Funding organizations had no role in study conduct or reporting.

We screened 206 individuals and enrolled 24 participants. This included 14 participants in the pre‐BTACT phase and 10 participants after implementing the BTACT. The mean age was 35 years; most participants were female (67%), and there were no significant differences in RPQ scores or blood biomarker levels before versus after BTACT administration (Table S1).

All 10 participants successfully completed the BTACT in full. The median (IQR) BTACT composite *z*‐score was −0.4863 (−1.075 to −0.1561). The median (IQR) length of time for BTACT completion was 15.67 (2.43) min. ED length of stay for individuals who completed the BTACT was a median of 4.12 h versus 4.04 h for those in the pre‐BTACT group (Figure 1). The median increase in ED length of stay associated with BTACT completion was 0.085 h (95% CI −0.820 to 1.650; *p* = 0.64), or 5.1 min.

**FIGURE 1:**
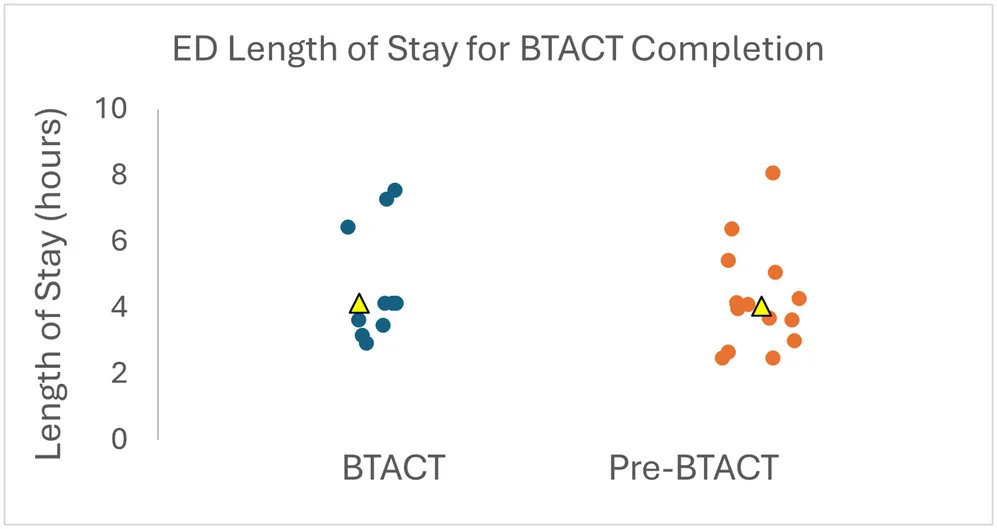
Dot plot of length of stay of participants who completed the BTACT versus participants who did not complete the BTACT (Pre‐BTACT). Yellow triangles represent median values.

Negative correlation of moderate effect size between RPQ and BTACT scores did not reach statistical significance (*r* = −0.4712, *p* = 0.17; Figure S2). BTACT score was not strongly associated with RPQ‐Cognitive sub‐score (*r* = −0.1846, *p* = 0.61) or years of education (*r* = −0.1506, *p* = 0.68). Correlation effect sizes between BTACT score and biomarker levels ranged from *r* = 0.09 (Tau) to *r* = −0.5152 (GFAP) but were not statistically significant (Figure S3).

Identifying feasible cognitive testing instruments for ED use is important to advance clinical care and research among patients with mTBI. Our findings demonstrate in‐person BTACT assessment in a small sample of participants with mTBI is feasible and practical in the ED as a quantitative cognitive assessment for research, taking an average of < 16 min per person, and not meaningfully impacting overall ED length of stay.

Other cognitive tests have been used in ED TBI research, including the Immediate Post‐concussion Assessment and Cognitive Testing (ImPACT) test and the Cambridge Neuropsychological Test Automated Battery (CANTAB) battery [9, 10], but these proprietary softwares are costly and not widely used.

In contrast, the BTACT has several advantages; it is freely available in paper format and referenced to a large normative sample for population‐level inferences. For example, our median BTACT *z*‐score was −0.46 (0.46 standard deviations below the normative mean, or ~32nd percentile), indicating participants had a modest shift towards below average cognitive function, consistent with mTBI [9]. Our research staff felt training burden was low (they completed two 30‐min supervised test sessions) and testing was easy to administer by following the administration manual [8]. We encountered no staff or patient acceptability concerns. Disadvantages of the BTACT include that it requires a staff member to administer and manually score the test, and it lacks an automated computerized version. BTACT administration could be interrupted for other ED testing, although we did not experience this.

Blood‐based biomarkers play an increasingly important role in clinical mTBI evaluation, and our understanding of biomarker relationships with clinical profiles including cognition is evolving. While we did not find statistically significant associations between BTACT and blood biomarker levels in these pilot data, observed correlations were in the expected negative direction and with moderate effect size for GFAP and UCHL1, suggesting more study is needed to determine how they jointly reflect brain injury burden.

This study has associated limitations. Small sample size limited statistical power of between‐group and correlation analyses, although the moderate effect size correlations support larger well‐powered studies to interrogate these relationships. In this observational pre‐post study design, we cannot draw causal conclusions or rule out non‐measured confounding variables, particularly in BTACT versus pre‐BTACT group comparisons; further validation is needed. Enrollment was limited by research personnel availability (0800–2000), low enrollment rate, and excluding people with prior brain injury or disease; BTACT feasibility may differ in excluded groups or times‐of‐day. These single‐center analyses may not be indicative of ED populations in other regions. This study assessed BTACT feasibility as a research tool for cognitive phenotyping rather than clinical utility; future studies should evaluate prognostic and clinical applications.

In conclusion, we believe the BTACT is a feasible ED mTBI cognitive assessment tool. Emergency medicine researchers may seek to adopt this as a useful cognitive assessment in ED‐based clinical research.

## Author Contributions

**David J. Barton:** study concept and design, acquisition of the data, analysis and interpretation of the data, drafting of the manuscript, critical revision of the manuscript for important intellectual content, statistical expertise, and acquisition of funding. **Charles F. Rudolph:** acquisition of the data, analysis and interpretation of the data, drafting of the manuscript, critical revision of the manuscript for important intellectual content. **Ekaterina K. Kalinina:** acquisition of the data. **Keshav Reddy:** acquisition of the data, critical revision of the manuscript for important intellectual content. **Natalee Gallo:** acquisition of the data. **Harry Morford:** acquisition of the data. **Xuemei Zeng:** acquisition of the data, analysis and interpretation of the data. **Thomas K. Karikari:** acquisition of the data, analysis and interpretation of the data. **Maria L. Pacella‐LaBarbara:** analysis and interpretation of the data, critical revision of the manuscript for important intellectual content. **Clifton W. Callaway:** study concept and design, analysis and interpretation of the data. **Amy K. Wagner:** study concept and design, analysis and interpretation of the data, critical revision of the manuscript for important intellectual content.

## Funding

This work was supported by the Emergency Medicine Foundation; National Institutes of Health through the Clinical and Translational Sciences Institute (CTSI) at the University of Pittsburgh (Grant Number UL1‐TR‐001857).

## Conflicts of Interest

The authors declare no conflicts of interest.

## Supporting information


**Figure S1:** Study sample flow chart of screened, excluded, eligible, and enrolled individuals.

**Figure S2:** Scatter plots and Spearman correlation coefficients between BTACT *z*‐scores and RPQ total score and RPQ‐Cognitive Sub‐score.

**Figure S3:** Scatter plots and Spearman correlation coefficients between BTACT *z*‐scores and blood biomarker levels.

**Table S1:** Participant demographics and clinical characteristics.

## Data Availability

The data that support the findings of this study are available on request from the corresponding author. The data are not publicly available due to privacy or ethical restrictions.
