# Supplementary material for: Emergency Department Feasibility of the Brief Test of Adult Cognition by Telephone (BTACT) for Research in Mild Traumatic Brain Injury
Source: Acad Emerg Med. 2026 Sep 29;33(10):e70420. doi: 10.1111/acem.70420 (PMC13624281; doi:10.1111/acem.70420)
Supplement: Supplementary file 1 [file ACEM-33-0-s002.pdf]

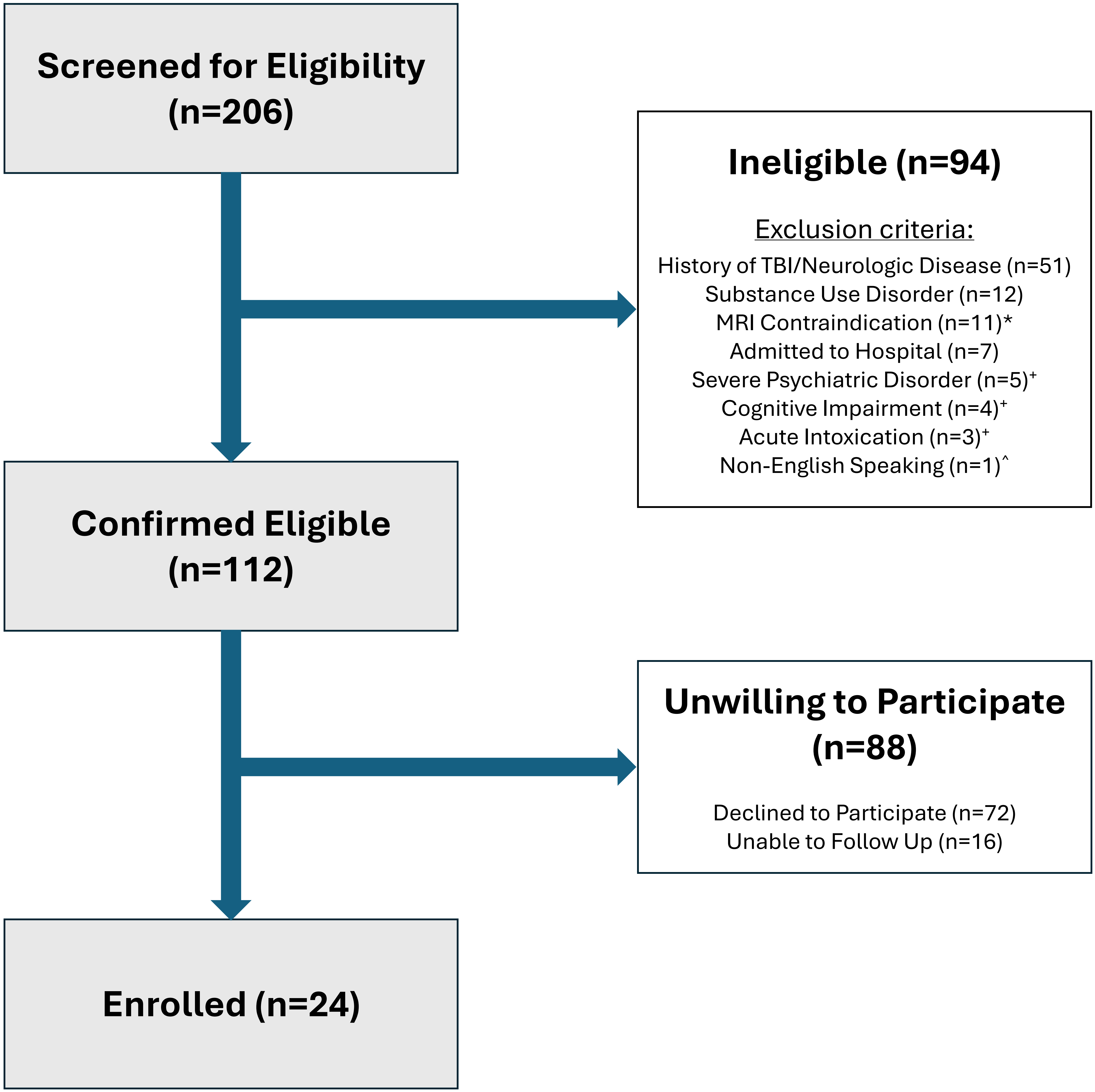

*Brain MRI was part of the overall research study, but MRI data (not reported here) are beyond the scope of the current analysis.
+Due to these conditions, patients were unable to consent to participate in the research.
^ BTACT instrument is not available or validated in non-English versions.
Other exclusion criteria were pregnancy (n=0) and incarceration (n=0).
