# Supplementary material for: Emergency Department Feasibility of the Brief Test of Adult Cognition by Telephone (BTACT) for Research in Mild Traumatic Brain Injury
Source: Acad Emerg Med. 2026 Sep 29;33(10):e70420. doi: 10.1111/acem.70420 (PMC13624281; doi:10.1111/acem.70420)
Supplement: Supplementary file 2 [file ACEM-33-0-s003.pdf]

Supplemental Table 1. Participant Demographics and Clinical Characteristics

Characteristic	BTACT group (n=10)	Pre-BTACT group (n=14)	All Participants (n=24)
Age (median [IQR]), y	29.5 (25.75, 43)	32.5 (25.5, 40.75)	30 (25, 41.75)
Sex, n (%)			
Male	3 (30%)	5 (35.71%)	8 (33.33%)
Female	7 (70%)	9 (64.29%)	16 (66.67%)
Years of education (median [IQR])	16 (13.25, 16)	16 (15.75, 18)	16 (14.25, 17.5)
Method of Injury, n (%)			
Fall	1 (10%)	6 (42.86%)	7 (29.17%)
Struck by Object	1 (10%)	2 (14.29%)	3 (12.50%)
MVA	8 (80%)	4 (28.57%)	12 (50%)
Sports-Related Incident	0 (0%)	1 (7.14%)	1 (4.17%)
Passed Out	0 (0%)	1 (7.14%)	1 (4.17%)
Head CT performed, n (%)	10 (100%)	11 (78.6%)	21 (87.5%)
Blood laboratory studies ordered, n (%)	4 (40%)	8 (57.1%)	12 (50%)
RPQ score (median, [IQR])	20.5 (13.5, 30.5)	19 (12, 32.5)	19 (12, 32.25)
RPQ-Cognitive sub-score (median, [IQR])	5 (1, 7.75)	6 (4, 7)	5.5 (3.25, 7)
Biomarker levels (median [IQR]), ng/mL			
GFAP	106.28 (50.71, 127.15)	92.48 (72.55, 130.29)	102.28 (58.77, 131.45)
UCH-L1	26.51 (15.87, 37.81)	24.12 (11.61, 38.68)	25.18 (14.87, 38.85)
NfL	6.36 (3.68, 6.56)	6.23 (4.41, 7.73)	6.33 (3.69, 7.26)
Tau	3.25 (2.94, 4.57)	4.85 (3.20, 6.88)	3.62 (3.00, 6.34)

Biomarker data was available for 23 of the 24 participants. One sample was unreadable and therefore excluded from data analysis. Years of education data was available for 22 of the 24 participants.
